# Three-Armed Trials Including Placebo and No-Treatment Groups May Be Subject to Publication Bias: Systematic Review

**DOI:** 10.1371/journal.pone.0020679

**Published:** 2011-05-31

**Authors:** Yun Hyung Koog, Seo Ryang We, Byung-Il Min

**Affiliations:** 1 Honam Research Center, Medifarm Hospital, Suncheon, Republic of Korea; 2 Department of East-West Medicine, Graduate School, Kyung Hee University, Seoul, Republic of Korea; 3 Department of Physiology, School of Medicine, Kyung Hee University, Seoul, Republic of Korea; Marienhospital Herne - University of Bochum, Germany

## Abstract

**Background:**

It has been argued that placebos may not have important clinical impacts in general. However, there is increasing evidence of a publication bias among trials published in journals. Therefore, we explored the potential for publication bias in randomized trials with active treatment, placebo, and no-treatment groups.

**Methods:**

Three-armed randomized trials of acupuncture, acupoint stimulation, and transcutaneous electrical stimulation were obtained from electronic databases. Effect sizes between treatment and placebo groups were calculated for treatment effect, and effect sizes between placebo and no-treatment groups were calculated for placebo effect. All data were then analyzed for publication bias.

**Results:**

For the treatment effect, small trials with fewer than 100 patients per arm showed more benefits than large trials with at least 100 patients per arm in acupuncture and acupoint stimulation. For the placebo effect, no differences were found between large and small trials. Further analyses showed that the treatment effect in acupuncture and acupoint stimulation may be subject to publication bias because study design and any known factors of heterogeneity were not associated with the small study effects. In the simulation, the magnitude of the placebo effect was smaller than that calculated after considering publication bias.

**Conclusions:**

Randomized three-armed trials, which are necessary for estimating the placebo effect, may be subject to publication bias. If the magnitude of the placebo effect is assessed in an intervention, the potential for publication bias should be investigated using data related to the treatment effect.

## Introduction

The “powerful placebo” [1] was widely accepted until recently, when consecutive reviews of the placebo effect were published [2]–[4]. In these reviews, authors defined the placebo effect as the difference in outcome measures between placebo and no-treatment groups [5]. All possible randomized trials with three arms (i.e., an active treatment group, a placebo group, and a no-treatment group) were rigorously collected. The authors found that although the effect varied from large to non-existent, the placebo generally did not have a powerful impact in clinical situations [4].

Because such conclusions were based on publicly reported clinical trials, trials used for analysis should be unbiased. However, there have been concerns over publication bias [6]–[10], where small studies with negative results in an active group would be less likely to be published. In a recent study on antidepressant agents [11], 37 of 38 trials that were deemed positive by the Food and Drug Administration of the United States were published in journals, whereas only 3 of 36 trials with negative results were published. In fact, 11 of 36 trials with negative results were published in journals in a way that conveyed a positive outcome.

If three-armed trials that include placebo and no-treatment groups are subject to publication bias, the conclusion for the placebo effect might be misleading. To address the publication bias in three-armed trials, we investigated two datasets on active treatment versus placebo groups and placebo versus no-treatment groups. Because acupuncture has been a hot-button issue in discussions of the placebo effect [12], [13], our study focuses on acupuncture and its relevant interventions (i.e., acupoint stimulation and transcutaneous electrical nerve stimulation (TENS)).

## Methods

### Search strategy

All trials were identified by searching randomized trials using the search terms pertaining to each treatment via MEDLINE (PubMed), EMBASE (or SCOPUS), and the Cochrane Central Register of Controlled Trials from their inception through October 2009. For example in PubMed, we used terms for acupuncture [“acupuncture” OR “electroacupuncture”], terms for acupoint stimulation [“acupressure” OR “acustimulation” OR “acupoint stimulation” OR “acupoint massage” OR “capsicum plaster” OR “transcutaneous electrical stimulation” OR “functional electrical stimulation”], and terms for TENS [“transcutaneous electrical stimulation” OR “transcutaneous electrical nerve stimulation” OR “TENS”], with limits to randomized controlled trials and humans. We used EMBASE for acupuncture and SCOPUS for the other two interventions because the availability of EMBASE expired at Asan Medical Library during our search. We defined acupoint simulation as any treatment that simulates the traditional acupuncture points without penetrating human skin.

### Selection criteria

The titles and abstracts of all resulting papers were read by two independent reviewers. However, those retrieved for TENS via the Cochrane Central Register of Controlled Trials database were read jointly. We then independently selected trials that included the following: (1) a randomized clinical trial; (2) a group where an intervention was pragmatically labeled as placebo; and (3) comparison of treatment, placebo, and no-treatment groups under identical conditions in one trial. However, we found only four [14]–[17], four [18]–[21], and zero trials with binary outcomes for acupuncture, acupoint stimulation, and TENS, respectively. Because regression-based tests are reported to have low statistical power for 10 or fewer trials [22], we decided to present the results of trials with continuous outcomes.

### Data extraction

Prior to data extraction, we prepared a protocol. First, we attempted to select the main outcome that was considered primary or was used for power calculation. When the above conditions were not fulfilled, there were two methods we could choose: (1) selecting the outcome on which the conclusion was based or (2) choosing the outcome reported first in the table or figure. When we examined the data of a previous report [4] using 31 eligible trials that did not explicitly report the main outcome, the former method resulted in 27 matches, whereas the latter resulted in 23 matches. Therefore, we extracted data using the former method. Second, we attempted to extract end-point data, because 52 eligible trials reported end-point data, whereas only 15 reported data on change from baseline. If such data were not available, the data on change from baseline were used. Third, we attempted to extract data evaluated at the end of the treatment, because most trials reported data assessed at the end of the treatment. We (KYH and WSR) then independently extracted data from eligible trials and referenced the previous reviews [4], [13] in open discussion. When necessary, we contacted the corresponding authors of included trials.

However, we met one problem in a trial [23] where standard deviations could not be obtained. Because the outcome used in this trial was unique within all eligible trials, we extracted data on the outcome used in a previous report [4].

In addition, we (KYH and WSR) independently extracted information on disease type and data type, as well as methodological characteristics (i.e., allocation concealment, assessor blinding, attrition rate, and intention-to-treat analysis). Allocation concealment was considered adequate if researchers responsible for patient selection could not predict the next treatment for a patient. Assessor blinding was considered adequate if outcome measures of interest were evaluated by researchers blinded to the treatment allocation or by objective instruments. Attrition rate was considered adequate if the flow of the patients' dropout throughout the trial was explicitly stated, and the attrition rate of all randomized patients who were assessed at baseline was below 15%. Intention-to-treat analysis was considered adequate if all randomized patients who were assessed at baseline were included in the analysis.

### Data synthesis

In each trial, we calculated effect sizes (standardized mean differences) between the active treatment and placebo groups and between the placebo and no-treatment groups. The effect sizes between active treatment and placebo groups were defined as “treatment effect” and those between placebo and no-treatment as “placebo effect”. We excluded trials from the calculations that reported only median and range because estimation from median and range might produce bias [24]. Indeed, the effect size calculated from median and range was overestimated in our previous study [25]. We also excluded trials that were clear outliers. To do this, we performed a test based on the blocked adaptive computationally efficient outliers nominator algorithm [26], with a significance level of 0.15.

### Identification of small study effects

We used four methods to address small study effects, where the smaller studies in a meta-analysis show larger treatment effects. First, we considered trials with more than 200 patients at baseline in two relevant arms as “large” trials and trials with fewer than 200 patients as “small” trials [27]. For example, when we considered a trial where 300 patients were randomized to an active treatment group (n = 150), a placebo group (n = 75), and a no-treatment group (n = 75), it was classified as a large trial for the treatment effect and as a small trial for the placebo effect. We then calculated the effect sizes of large and small trials separately using a random effects model [28] and derived the differences between the effect sizes of large and small trials. The p value was based on an interaction test, which is defined as the difference in effect sizes divided by the standard error of the difference [29]. For summary estimates, we combined all differences between large and small trials using a random effects model. Second, we drew a contour-enhanced funnel plot [30]. In this study, a plot was divided into areas of significance (two-sided P≤0.05) and areas of non-significance (two-sided P>0.05). Thirdly, we evaluated funnel plot asymmetry using the asymmetry coefficient, which is defined as the difference in effect size per standard error increase [31]. To this end, we predicted a treatment or placebo effect from a weighted linear regression with the standard error as an independent variable. We then combined all asymmetry coefficients using a random effects model, crude and adjusted for methodological characteristics, clinical condition (pain or non-pain), and data type (subjective or objective outcome). Fourth, we performed an Egger's regression test [32].

### Identification of sources of small study effects

When the small study effects were detected, we performed two additional tests to exclude the other sources of small study effects (i.e., quality of methodological design and true heterogeneity) [32]. In the first test, we categorized trials by methodological characteristics and compared the pooled effect sizes between trials with or without characteristics based on an interaction test. Even if the small study effects were detected in only one treatment, we decided to show all three treatments to maintain the internal consistency of our study. In the second test, we investigated the causes of heterogeneity by univariate meta-regression using the following conditions: clinical conditions (pain or non-pain), disease duration (acute or chronic), cointervention (present or none), outcome type (objective or subjective), trial duration, and treatment session.

### Simulation

First, we estimated two different effect sizes for the treatment and placebo effect in each intervention: (1) pooled effect sizes from all identified trials and (2) effect sizes predicted at standard error  = 0 for hypothetical trials of infinite size [31], [33] from random effects meta-regression analysis with the standard error as an independent variable [27], [32]. Second, we produced hypothetical trials that could be suppressed by publication bias using a non-parametric trim and fill analysis with a fixed effects model on original data for the treatment effect [34]. We estimated effect sizes for the treatment effect from identified trials and hypothetical trials. In hypothetical trials, we then assumed that active treatment was at least as effective as no-treatment. We added these trials to the original data on placebo versus no-treatment and estimated effect sizes for the placebo effect. Finally, we compared the three effect sizes for the treatment and placebo effect.

### Statistical analysis

For heterogeneity, we assessed the values of between-trial variance (τ^2^). The data are presented as the mean with 95% confidence interval. Microsoft Excel 2003 was used for interaction tests and STATA version 11.0 for all further analyses.

## Results

**Figure 1:**
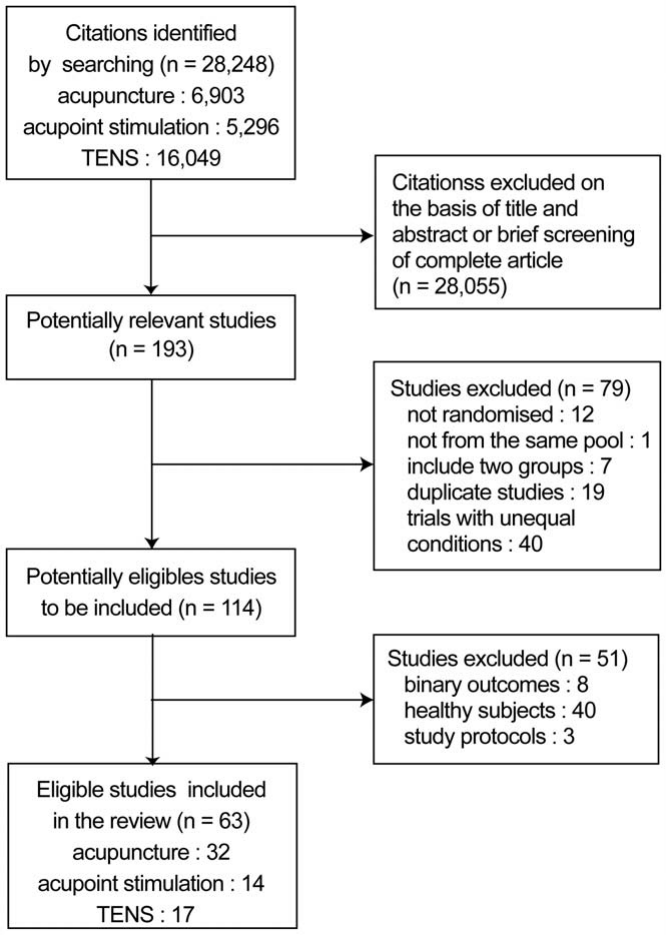
Study flow diagram.

Figure 1 describes the procedure for selecting eligible trials. We included 63 trials with continuous outcomes: 32 trials for acupuncture (Text S1), 14 trials for acupoint stimulation (text S2), and 17 trials for TENS (Text S3) (Table 1). Of these, the overall number of large trials with more than 200 patients in two relevant arms was small: 6 (18.8%) trials for treatment effect and 3 (9.4%) for placebo effect in acupuncture, 2 (11.8%) in TENS, and none in acupoint stimulation. In total, 3060 patients were included at baseline in the active treatment group, 2576 patients were included in the placebo group, and 2533 patients were included in the no-treatment group. In the eligible trials, many different clinical conditions were assessed. Acupuncture and TENS trials frequently studied pain-related disease, and acupoint stimulation trials frequently investigated nausea-related disease. Placebo type also varied within each intervention. Acupuncture needles that were normally inserted or minimally inserted at irrelevant points were commonly used as a placebo in acupuncture trials. Stimulation on irrelevant points was mostly used as the placebo in acupoint stimulation. Simulated TENS with electricity off was mostly used as the placebo in TENS.

**Table 1:** Characteristics of trials with continuous outcomes.

	Acupuncture (n = 32)	Acupoint stimulation (n = 14)	TENS (n = 17)
Number of large trials	6/3*	None	2
Total sample size†	2019/1601/1556	400/395/400	641/580/577
Clinical condition	Pain: 17	Pain: 2	Pain: 16
	Nausea: 1	Nausea: 6	
	Depression: 3	Insomnia: 2	
	Anxiety: 2	Others: 4	
	Others: 9		
Placebo type	Normal needling at irrelevant point: 11	No stimulation on relevant point: 2	TENS with no stimulation: 14
	Minimal needling at irrelevant point: 16	Stimulation on irrelevant point: 12	TENS with sub-threshold stimulation: 1
	No penetration: 2		TENS with non-segmental stimulation: 1
	Others: 2		
Treatment effect			
Effect size (95% CI)	0.41 (0.24 to 0.58)	0.64 (0.28 to 0.99)	0.30 (0.11 to 0.49)
Heterogeneity	τ^2^ = 0.16	τ^2^ = 0.34	τ^2^ = 0.06
Placebo effect			
Effect size (95% CI)	0.34 (0.19 to 0.49)	0.21 (0.07 to 0.35)	0.05 (−0.06 to 0.17)
Heterogeneity	τ^2^ = 0.10	τ^2^ = 0.00	τ^2^ = 0.00

CI  =  confidence interval.

*The earlier value is for treatment effect, and the latter for placebo effect.

† Numbers are values for active treatment, placebo, and no-treatment groups, respectively.

Of 63 eligible trials, 2 reported outcomes with median and range: one [35] for acupuncture and another [36] for acupoint stimulation. One trial [37] for TENS presented insufficient data (e.g., no patient number). One trial [38] for TENS was a clear outlier. Therefore, we excluded these trials from our analysis. Within the remaining trials, the summary treatment effects were 0.41 (0.24 to 0.58), 0.64 (0.28 to 0.99), and 0.30 (0.11 to 0.49) for acupuncture, acupoint stimulation, and TENS, respectively. The summary placebo effects were 0.34 (0.19 to 0.49), 0.21 (0.07 to 0.35), and 0.05 (−0.06 to 0.17), respectively. When heterogeneity was compared between the treatment and placebo effects, the placebo effects were less heterogeneous than the treatment effects in all interventions.

**Figure 2:**
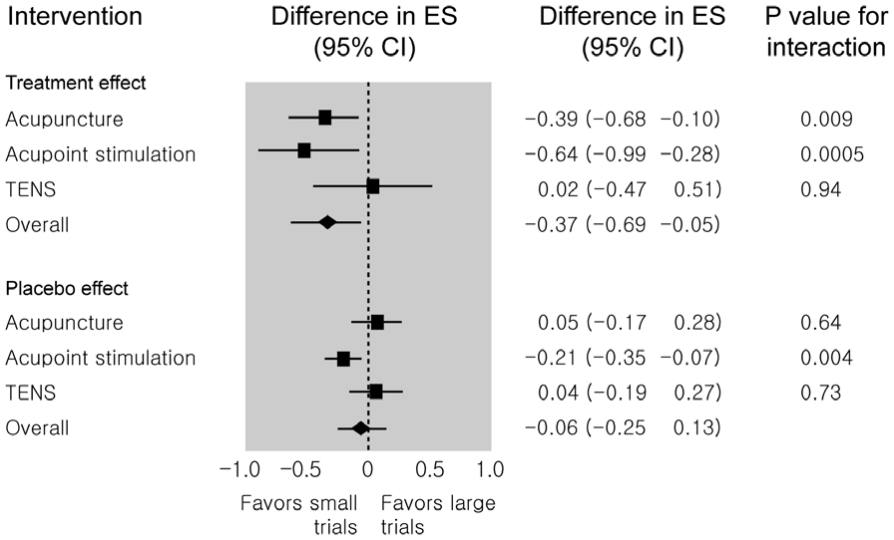
Difference in effect sizes between large trials with at least 100 patients per arm and small trials with fewer than 100 patients. ES  =  effect size.

When small and large trials were compared for treatment effect (Figure 2), the difference in effect sizes between large and small trials was statistically significant in acupuncture (P = 0.009) and acupoint stimulation (P = 0.0005). For acupuncture, small trials showed more benefits by 0.39 (0.10 to 0.68) in effect size than large trials, and for acupoint stimulation, more benefits by 0.64 (0.28 to 0.99) in effect size. However, there was no significant difference between small and large trials in TENS. The summary difference of −0.37 (−0.69 to −0.05) over the three interventions was statistically significant. When small and large trials were compared for placebo effect, a significant difference was found only in the acupoint stimulation (P = 0.004). The summary difference of −0.06 (−0.25 to 0.13) was not statistically significant.

**Figure 3:**
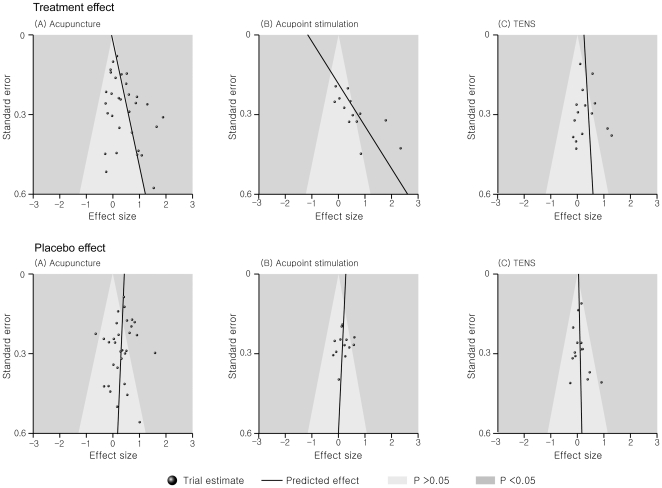
Contour-enhanced funnel plot including predicted lines from univariable meta-regression models.

Figure 3 presents the funnel plots, where predicted treatment or placebo effect lines (i.e., coefficient asymmetries) were included. For the treatment effect in acupuncture and acupoint stimulation, the left portion of the triangle was clearly missing when an imaginary triangle was drawn with the lowest standard error as a peak. In addition, the predicted treatment effect lines were not upright (P = 0.047 in acupuncture and P = 0.006 in acupoint stimulation) (Figure 3 and Table 2). However, the scatter plot of effect sizes in TENS was clearly symmetrical, and the predicted treatment effect line was upright (P = 0.975). The summary asymmetry coefficient was 2.48 (−0.54 to 5.50). Even when the summary asymmetry coefficient was adjusted for methodological characteristics, clinical condition, and data type, it was still similar to the crude value. For the placebo effect in the three interventions, the scatter plots of the effect sizes were clearly symmetrical and the predicted placebo effect lines were upright (P = 0.459, 0.638, and 0.683 for acupuncture, acupoint stimulation, and TENS, respectively) (Figure 3 and Table 2). The summary asymmetry coefficient was −0.11 (−0.99 to 0.78), and it did not differ after adjustment of methodological characteristics, clinical condition, and data type.

**Table 2:** Asymmetry coefficients.

	Treatment effect	Placebo effect
	Coefficient (95% CI)	Coefficient (95% CI)
	P value	P value
Acupuncture	1.80 (0.02 to 3.58)	−0.57 (−2.20 to 1.06)
	P = 0.047	P = 0.482
Acupoint stimulation	7.01 (2.52 to 11.49)	−0.67 (−3.74 to 2.39)
	P = 0.006	P = 0.638
TENS	0.04 (−2.44 to 2.52)	0.25 (−1.03 to 1.53)
	P = 0.975	P = 0.683
Summary coefficient	2.48 (−0.54 to 5.50)	−0.11 (−0.99 to 0.78)

CI  =  confidence interval.

Table 3 shows the results of Egger's regression tests. For the treatment effect, bias was present in acupuncture (P = 0.012) and acupoint stimulation (P = 0.005), although no bias was found in TENS (P = 0.716). For the placebo effect, no significant bias was found in any of the interventions (P = 0.376, 0.607, and 0.665 for acupuncture, acupoint stimulation, and TENS, respectively).

**Table 3:** Egger's regression tests.

	Treatment effect	Placebo effect
	Coefficient (95% CI)	Coefficient (95% CI)
	P value	P value
Acupuncture	1.99 (0.47 to 3.51)	−0.64 (−2.09 to 0.81)
	P = 0.012	P = 0.376
Acupoint stimulation	6.84 (2.53 to 11.16)	−0.67 (−3.48 to 2.13)
	P = 0.005	P = 0.607
TENS	0.34 (−1.62 to 2.30)	0.25 (−0.96 to 1.46)
	P = 0.716	P = 0.665

CI  =  confidence interval.

Table 4 presents the pooled treatment effects of three interventions categorized by methodological characteristics. P values for the interaction test did not show any significant differences between trials in any of the three interventions. When the causes of heterogeneity were examined, no factor was associated with the effect sizes in acupuncture or acupoint stimulation.

**Table 4:** Treatment effect of trials with or without methodological characteristics.

	Acupuncture			Acupoint stimulation			TENS		
	Effect size (95% CI)		P*	Effect size (95% CI)		P*	Effect size (95% CI)		P*
**Allocation concealment**									
Yes	0.28	(0.11 to 0.46)	0.16	0.94	(−0.39 to 2.26)	0.59	0.11	(−0.06 to 0.29)	0.06
No/unclear	0.55	(0.21 to 0.89)		0.56	(0.21 to 0.91)		0.41	(0.16 to 0.65)	
**Assessor blinding**									
Yes	0.42	(0.20 to 0.65)	0.90	0.72	(0.04 to 1.41)	0.75	0.19	(−0.07 to 0.45)	0.29
No/unclear	0.40	(0.12 to 0.68)		0.59	(0.15 to 1.03)		0.39	(0.12 to 0.67)	
**Attrition rate**									
Good	0.42	(0.22 to 0.62)	0.94	0.47	(−0.05 to 0.99)	0.39	0.11	(−0.08 to 0.30)	0.09
Bad	0.40	(0.04 to 0.77)		0.78	(0.31 to 1.26)		0.37	(0.14 to 0.60)	
**Intention-to-treat analysis**									
Yes	0.30	(0.08 to 0.51)	0.14	1.32	(−0.66 to 3.31)	0.44	0.12	(−0.27 to 0.52)	0.36
No/unclear	0.56	(0.28 to 0.84)		0.53	(0.18 to 0.88)		0.33	(0.12 to 0.54)	

CI  =  confidence interval.

*P values are based on interaction test.

**Figure 4:**
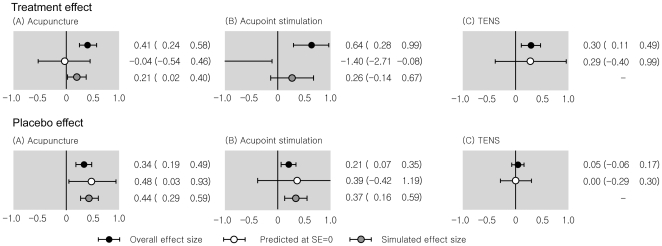
Results of effect sizes combined over all trials, effect sizes predicted for trials from random effects meta-regression analysis with standard error  = 0, and effect sizes simulated on data from nonparametric trim and fill analysis. SE  =  standard error.

Figure 4 shows the results of the pooled effect sizes of all eligible trials, the predicted effect sizes for hypothetical trials with infinite size, and the simulated effect sizes for data from non-parametric trim and fill analysis. For the treatment effect (Figure 4A and B), the effect sizes combined over eligible trials were greater than those predicted for hypothetical trials with infinite size or those simulated on data from nonparametric trim and fill analysis. For the placebo effect (Figure 4A and B), the effect sizes combined over eligible trials were smaller than those simulated on data from non-parametric trim and fill analysis, although they were included within the range of the 95% confidence interval for hypothetical placebo effect from meta-regression with standard error  = 0.

## Discussion

In this study on three-armed trials for placebo effect, we found that small trials showed a greater effect than large trials (i.e., small study effects) when examining the treatment effect for acupuncture and acupoint stimulation, defined by the effect size between active and placebo groups. We did not find any such tendency in the placebo effect for the three interventions, defined by the effect size between placebo and no-treatment groups. In further analysis, the small study effects in acupuncture and acupoint stimulation did not appear to be related to trial methodology or true heterogeneity, thus indicating publication bias.

It is surprising that some three-armed trials may be published according to the significance of an active treatment group. If trials with a significantly greater effect of an active treatment compared with a placebo are more likely to be published, the magnitude of the placebo effect may be seriously biased. In fact, when the missing trials were considered, the summary treatment effects for acupuncture and acupoint stimulation decreased from those combined over all eligible trials (Figure 4). In contrast, the summary placebo effects for acupuncture and acupoint stimulation increased from those pooled over all eligible trials (Figure 4). Consequently, publication bias distorted the results of meta-analyses based on identified trials for both effects.

However, it should be noted that the magnitude of the placebo effect cannot be accurately predicted, because excellent statistical analyses cannot predict missing trials accurately. In fact, a trim and fill analysis using a random effects model detected no missing trials in three interventions. Although missing trials are identified by some analyses, the magnitude of placebo effect cannot be easily conjectured. In the simulation, we assumed that active treatment was at least as effective as no-treatment. However, this assumption cannot always be applied in general situations. Because active treatment may be superior to no-treatment in most situations [39], the magnitude of the placebo effect will be much greater than that predicted in the simulation. Therefore, we are not sure, at present, whether the placebo effect from meta-regression with standard error  = 0 can predict the placebo effect that was recalculated after considering publication bias, although the former predicted the latter in our simulation.

Previous reviews [2]–[4] have shown that placebos may not have important clinical impacts in general. This finding led some researchers to conclude that the concept of a “powerful placebo” remains groundless [40]. However, our finding implies that the small overall placebo effect might be produced by publication bias. Because publication bias is dependent on the significance of treatment effect, trials published in journals are more likely to have a relatively smaller placebo effect. If such trials are combined, the overall placebo effect would be small.

Previous reviews [4], [13] have also shown that when placebos were examined in acupuncture trials with high quality, they were associated with greater effect in some situations and with non-existing effect in other situations. However, our finding implies that the variable magnitude of the placebo effect may be secondary to the natural process of publication. For example, if one intervention is developed as a new therapy, trials with greater effect of intervention begin to be published. At this time, the heterogeneity of the placebo effect would be small. However, trials with a smaller or negative effect will be published in the future. In this case, the magnitude of the placebo effect begins to be variable. To confirm this, we categorized acupuncture trials by publication year. Interestingly, as time passed, the value of τ^2^ for the placebo effect gradually increased from 0.00 to 0.10 with a shape of ∼.

Previous reviews [2]–[4] have found that the placebo effect on pain-related clinical conditions was great. We did not address this point in our study, but we did address other questions regarding whether methodological characteristics or some other factors were associated with publication bias. We found that none were associated with publication bias. However, we only investigated three interventions. Furthermore, we could not extract diverse factors from trials of each intervention (e.g., all TENS trials were focused on pain-related conditions). Therefore, all interventions should be investigated to determine whether certain factors are associated with publication bias.

Although a previous review [4] and our study investigated the placebo effect using the same criteria, interpretations were very different. The discrepancies can be explained in several ways.

First, we analyzed many other trials, including the most recent ones. We reviewed all randomized trials, even if the abstract was not in the web databases. Surprisingly, this simple search strategy yielded more trials than the updated review that used complex search strategies aimed at detecting all three groups in one trial. When trials published up to March 2008 were considered, we consequently included seven more acupuncture trials [41]–[47], two more acupoint stimulation trials [48], [49], and four more TENS trials [37], [50]–[52] than the previous review.

Second, we investigated two datasets on the treatment and placebo effects of each individual intervention. Using two datasets, we attempted to study whether three-armed trials for the placebo effect were biased. We found that the placebo effect should be explored after examining the potential for bias on the treatment effect. Meanwhile, the previous review studied only one dataset on the placebo effect. The previous review also investigated the potential for bias. However, our finding suggests that it is difficult to find any bias in such early investigations of data related to the placebo effect.

In our study, we attempted to prove publication bias. To this end, we tried to review all randomized trials and thus included many relevant three-armed trials. However, reviewing all randomized trials is labor-intensive and time-consuming. Unfortunately, we may have missed some relevant trials. In addition, we did not use several potential sources to identify further trials. First, we did not consult the existing relevant review [4]. When our study was compared with the previous review [4], we found that one trial [53] was not included in our study. Second, we did not search the public trial registries, such as ClinicalTrials.gov (http://www.clinicaltrials.gov/) or the International Standard Randomized Controlled Trial Number Web site (http://www.controlled-trials.com/isrctn/). It is possible that three-armed trials might be reported as two-armed trials in journals for many reasons (e.g., authors' performance). Unless such trials explicitly report this point, they cannot be easily identified without searching the clinical trials registers. The failure to use means at our disposal to identify additional trials represents a limitation of this study.

In this study, we did not fully address the issue of publication bias. Because there are no definitive methods to evaluate publication bias [54], we assessed small study effects and then excluded two potential sources of small study effects (i.e., quality of methodological design and true heterogeneity). However, some researchers [55] may wonder whether the small study effects were associated with real treatment effects. It is possible that patients at high risk of disease in smaller trials could have received substantial benefits from interventions. However, when we examined acupuncture trials reporting pain intensity, patients with more severe pain did not receive increased benefits (P = 0.50). Other researchers [32] may wonder whether interventions have been implemented less thoroughly in larger trials, thus resulting in more positive results than in smaller trials. When acupuncture trials were considered as an example, this appeared to be unlikely because relatively larger trials [56]–[58] utilized semi-individualized treatments, whereas small trials [41], [44]–[46] only utilized standardized treatments.

We think that our findings have laid the groundwork for debate on the use of placebos in clinical practice. Clinical evidence in support of the placebo effect has been accumulated in a wide range of conditions [59]–[63]. However, the evidence has been discounted because it was derived from randomized trials that did not include a no-treatment group [64]. In contrast, previous reviews [2]–[4] addressing this defect argued that the placebo effect was limited in general. We revealed that this argument might be misleading. To sum up, the placebo effect appears to be a common phenomenon. Therefore, ethical guidelines for the use of placebos should be discussed [65]. We also think that our findings provide different viewpoints on the placebo effect. Two previous reviews [2], [3] concluded that the greater placebo effect was associated with pain-related clinical conditions, and a recent study [4] added that physical placebo interventions were also associated. However, according to our findings, the placebo effect for TENS was not great in pain-related conditions. Therefore, our findings indicate that analyzing the placebo effect as categorized by intervention is also important.

### Conclusions

Consequently, randomized three-armed trials necessary for estimating the placebo effect were published in journals according to the significance of an active treatment group in some interventions. Publication bias distorted results for the placebo effect in meta-analyses based soley on identified trials. Therefore, if the magnitude of the placebo effect is being assessed in some interventions, the potential for publication bias should be investigated in data related to the treatment effect.

## Supporting Information

Text S1 Trials for acupuncture.
(DOC)

Text S2 Trials for acupoint stimulation.
(DOC)

Text S3 Trials for transcutaneous electrical nerve stimulation.
(DOC)
