# Supplementary material for: Three-Armed Trials Including Placebo and No-Treatment Groups May Be Subject to Publication Bias: Systematic Review
Source: PLoS One. 2011 May 31;6(5):e20679. doi: 10.1371/journal.pone.0020679 (PMC3105112; doi:10.1371/journal.pone.0020679)
Supplement: Text S1 — Trials for acupuncture. (DOC) [file pone.0020679.s001.doc]

Text S1. Trials for acupuncture

1. Lee SH, Lee BC (2009). Electroacupuncture relieves pain in men with chronic prostatitis/chronic pelvic pain syndrome: three-arm randomized trial. Urology 73(5): 1036-41.

2. Sertel S, Herrmann S, Greten HJ, Haxsen V, El-Bitar S, et al. (2009) Additional use of acupuncture to NSAID effectively reduces post-tonsillectomy pain. Eur Arch Otorhinolaryngol 266(6): 919-25.

3. Grecco CM, Kao AH, Maksimowicz-McKinnon K, Glick RM, Houze M, et al. (2008) Acupuncture for systemic lupus erythematosus: a pilot RCT feasibility and safety study. Lupus 17(12): 1108-16.

4. Avis NE, Legault C, Coeytaux RR, Pian-Smith M, Shifren JL, et al. (2008) A randomized, controlled pilot study of acupuncture treatment for menopausal hot flashes. Menopause 15(6): 1070-8.

5. Facco E, Liguori A, Petti F, Zanette G, Coluzzi F, et al. (2008) Traditional acupuncture in migraine: a controlled randomized study. Headache 48: 398-407.

6. Freire AO, Sugai GC, Chrispin FS, Togeiro SM, Yamamura Y, et al. (2007) Treatment of moderate obstructive sleep apnea syndrome with acupuncture: a randomised, placebo-controlled pilot trial. Sleep Med 8(1): 43-50.

7. Foster NE, Thomas E, Barlas P, Hill JC, Young J, et al. (2007) Acupuncture as an adjunct to exercise based physiotherapy for osteoarthritis of the knee: randomised controlled trial. BMJ 1335(7617): 436.

8. Allen JJ, Schnyer RN, Chambers AS, Hitt SK, Moreno FA, et al. (2006) Acupuncture for depression: a randomized controlled trial. J Clin Psychiatry 67(11): 1665-73.

9. Gioia L, Cabrini L, Gemma M, Fiori R, Fasce F, et al. (2006) Sedative effect of acupuncture during cataract surgery: prospective randomized double-blind study. J Cataract Refract Surg 32(11): 1951-4.

10. Cabrini L, Gioia L, Gemma M, Melloni G, Carretta A, et al. (2006) Acupuncture for diagnostic fiberoptic bronchoscopy: a prospective, randomized, placebo-controlled study. Am J Chin Med 34(3): 409-15.

11. Scharf HP, Mansmann U, Streitberger K, Witte S, Krämer J, et al. (2006) Acupuncture and knee osteoarthritis: a three-armed randomized trial. Ann Intern Med 145(1): 12-20.

12. Ziaei S, Hajipour L (2006) Effect of acupuncture on labor. Int J Gynaecol Obstet 92(1): 71-2.

13. Brinkhaus B, Witt CM, Jena S, Linde K, Streng A, et al. (2006) Acupuncture in patients with chronic low back pain: a randomized controlled trial. Arch Intern Med 166(4): 450-7.

14. Melchart D, Streng A, Hoppe A, Brinkhaus B, Witt C, et al. (2005) Acupuncture in patients with tension-type headache: randomised controlled trial. BMJ 331(7513): 376-82.

15. Witt C, Brinkhaus B, Jena S, Linde K, Streng A, et al. (2005) Acupuncture in patients with osteoarthritis of the knee: a randomised trial. Lancet 366(9480): 136-43.

16. Linde K, Streng A, Jürgens S, Hoppe A, Brinkhaus B, et al. (2005) Acupuncture for patients with migraine: a randomized controlled trial. JAMA 293(17): 2118-25.

17. Schuler MS, Durdak C, Hosl NM, Klink A, Hauer KA, et al. (2005) Acupuncture treatment of geriatric patients with ischemic stroke: a randomized, double-controlled, single-blind study. J Am Geriatr Soc 53(3): 549-50.

18. Johnstone PA, Bloom TL, Niemtzow RC, Crain D, Riffenburgh RH, et al. (2003) A prospective, randomized pilot trial of acupuncture of the kidney-bladder distinct meridian for lower urinary tract symptoms. J Urol 169(3): 1037-9.

19. Rösler A, Otto B, Schreiber-Dietrich D, Steinmetz H, Kessler KR (2003) Single-needle acupuncture alleviates gag reflex during transesophageal echocardiography: a blinded, randomized, controlled pilot trial. J Altern Complement Med 9(6): 847-9.

20. Molsberger AF, Mau J, Pawelec DB, Winkler J (2002) Does acupuncture improve the orthopedic management of chronic low back pain--a randomized, blinded, controlled trial with 3 months follow up. Pain 99(3): 579-87.

21. Leibing E, Leonhardt U, Köster G, Goerlitz A, Rosenfeldt JA, et al. (2002) Acupuncture treatment of chronic low-back pain -- a randomized, blinded, placebo-controlled trial with 9-month follow-up. Pain 96(1-2): 189-96.

22. Lin JG, Lo MW, Wen YR, Hsieh CL, Tsai SK, et al. (2002) The effect of high and low frequency electroacupuncture in pain after lower abdominal surgery. Pain 99(3): 509-14.

23. Medici TC, Grebski E, Wu J, Hinz G, Wüthrich B (2002) Acupuncture and bronchial asthma: a long-term randomized study of the effects of real versus sham acupuncture compared to controls in patients with bronchial asthma. J Altern Complement Med 8(6): 737-50; discussion 751-4.

24. Smith C, Crowther C, Beilby J (2002) Acupuncture to treat nausea and vomiting in early pregnancy: a randomized controlled trial. Birth 29(1): 1-9.

25. Kotani N, Kushikata T, Suzuki A, Hashimoto H, Muraoka M, et al. (2001) Insertion of intradermal needles into painful points provides analgesia for intractable abdominal scar pain. Reg Anesth Pain Med 26(6): 532-8.

26. Shen J, Wenger N, Glaspy J, Hays RD, Albert PS, et al. (2000) Electroacupuncture for control of myeloablative chemotherapy-induced emesis: A randomized controlled trial. JAMA 284(21): 2755-61.

27. Röschke J, Wolf C, Müller MJ, Wagner P, Mann K, et al. (2000) The benefit from whole body acupuncture in major depression. J Affect Disord 57(1-3): 73-81.

28. Gosman-Hedström G, Claesson L, Klingenstierna U, et al. (1998) Effects of acupuncture treatment on daily life activities and quality of life: a controlled, prospective, and randomized study of acute stroke patients. Stroke 29: 2100-8.

29. Allen JJB, Schyner RN, Hitt SK (1998) The efficacy of acupuncture in the treatment of major depression in women. Psychological Science 9: 397-401.

30. Sprott H (1998) Efficiency of acupuncture in patients with fibromyalgia. Clinical Bulletin of Myofascial Therapy 3: 37-43.

31. Tremeau ML, Fontanie-Ravier P, Teurnier F, Demouzon J (1992) [Protocol of cervical maturation by acupuncture]. J Gynecol Obstet Biol Reprod (Paris) 21(4): 375-80.

32. Helms JM (1987) Acupuncture for the management of primary dysmenorrhea. Obstet Gynecol 69(1): 51-6.
