# Supplementary material for: Three-Armed Trials Including Placebo and No-Treatment Groups May Be Subject to Publication Bias: Systematic Review
Source: PLoS One. 2011 May 31;6(5):e20679. doi: 10.1371/journal.pone.0020679 (PMC3105112; doi:10.1371/journal.pone.0020679)
Supplement: Text S2 — Trials for acupoint stimulation. (DOC) [file pone.0020679.s002.doc]

Text S2. Trials for acupoint stimulation

1. Yan T, Hui-Chan CW (2009) Transcutaneous electrical stimulation on acupuncture points improves muscle function in subjects after acute stroke: a randomized controlled trial. J Rehabil Med 41(5): 312-6.

2. Arai YC, Kato N, Matsura M, Ito H, Kandatsu N, et al. (2008) Transcutaneous electrical nerve stimulation at the PC-5 and PC-6 acupoints reduced the severity of hypotension after spinal anaesthesia in patients undergoing Caesarean section. Br J Anaesth 100(1): 78-81.

3. Shin HS, Song YA, Seo S (2007) Effect of Nei-Guan point (P6) acupressure on ketonuria levels, nausea and vomiting in women with hyperemesis gravidarum. J Adv Nurs 59(5): 510-9.

4. Maa SH, Tsou TS, Wang KY, Wang CH, Lin HC, et al. (2007) Self-administered acupressure reduces the symptoms that limit daily activities in bronchiectasis patients: pilot study findings. J Clin Nurs 16(4): 794-804.

5. Dibble SL, Luce J, Cooper BA, Israel J, Cohen M, et al. (2007) Acupressure for chemotherapy-induced nausea and vomiting: a randomized clinical trial. Oncol Nurs Forum 34(4): 813-20.

6. Roscoe JA, Matteson SE, Morrow GR, Hickok JT, Bushunow P, et al. (2005) Acustimulation wrist bands are not effective for the control of chemotherapy-induced nausea in women with breast cancer. J Pain Symptom Manage 29(4): 376-84.

7. Tsay SL (2004) Acupressure and fatigue in patients with end-stage renal disease-a randomized controlled trial. Int J Nurs Stud 41(1): 99-106.

8. Tsay SL, Rong JR, Lin PF (2003) Acupoints massage in improving the quality of sleep and quality of life in patients with end-stage renal disease. J Adv Nurs 42(2): 134-42.

9. Kober A, Scheck T, Greher M, Lieba F, Fleischhackl R, et al. (2002) Prehospital analgesia with acupressure in victims of minor trauma: a prospective, randomized, double-blinded trial. Anesth Analg 95(3): 723-7, table of contents.

10. Roscoe JA, Morrow GR, Bushunow P, Tian L, Matteson S (2002) Acustimulation wristbands for the relief of chemotherapy-induced nausea. Altern Ther Health Med 8(4): 56-7, 59-63.

11. Werntoft E, Dykes AK (2001) Effect of acupressure on nausea and vomiting during pregnancy. A randomized, placebo-controlled, pilot study. J Reprod Med 46(9): 835-9.

12. Chen ML, Lin LC, Wu SC, Lin JG (1999) The effectiveness of acupressure in improving the quality of sleep of institutionalized residents. J Gerontol A Biol Sci Med Sci 54(8): M389-94.

13. Wang B, Tang J, White PF, Naruse R, Sloninsky A, et al. (1997) Effect of the intensity of transcutaneous acupoint electrical stimulation on the postoperative analgesic requirement. Anesth Analg 85(2): 406-13.

14. O'Brien B, Relyea MJ, Taerum T (1996) Efficacy of P6 acupressure in the treatment of nausea and vomiting during pregnancy. Am J Obstet Gynecol 174(2): 708-15.
