# Supplementary material for: Three-Armed Trials Including Placebo and No-Treatment Groups May Be Subject to Publication Bias: Systematic Review
Source: PLoS One. 2011 May 31;6(5):e20679. doi: 10.1371/journal.pone.0020679 (PMC3105112; doi:10.1371/journal.pone.0020679)
Supplement: Text S3 — Trials for transcutaneous electrical nerve stimulation. (DOC) [file pone.0020679.s003.doc]

Text S3. Trials for transcutaneous electrical nerve stimulation

1. Solak O, Emmiler M, Ela Y, Dündar U, Koçoiullari CU, et al. (2009) Comparison of continuous and intermittent transcutaneous electrical nerve stimulation in postoperative pain management after coronary artery bypass grafting: a randomized, placebo-controlled prospective study. Heart Surg Forum 12(5): E266-71.

2. Emmiler M, Solak O, Kocogullari C, Dundar U, Ayva E, et al. (2008) Control of acute postoperative pain by transcutaneous electrical nerve stimulation after open cardiac operations: a randomized placebo-controlled prospective study. Heart Surg Forum 11(5): E300-3.

3. Tonella RM, Araújo S, Da Silva ÁMO (2006) Transcutaneous electrical nerve stimulation in the relief of pain related to physical therapy after abdominal surgery. Revista Brasileira de Anestesiologia 56(6): 630-42.

4. Hruby G, Ames C, Chen C, Yan Y, Sagar J, et al. (2006) Assessment of efficacy of transcutaneous electrical nerve stimulation for pain management during office-based flexible cystoscopy. Urology 67(5): 914-7.

5. Defrin R, Ariel E, Peretz C (2005) Segmental noxious versus innocuous electrical stimulation for chronic pain relief and the effect of fading sensation during treatment. Pain 115(1-2): 152-60.

6. Limoges MF, Rickabaugh B (2004) Evaluation of TENS during screening flexible sigmoidoscopy. Gastroenterol Nurs 27(2): 61-8.

7. Breit R, Van der Wall H (2004) Transcutaneous electrical nerve stimulation for postoperative pain relief after total knee arthroplasty. J Arthroplasty 19(1): 45-8.

8. Robinson R, Darlow S, Wright SJ, Watters C, Carr I, et al. (2001) Is transcutaneous electrical nerve stimulation an effective analgesia during colonoscopy? Postgrad Med J 77(909): 445-6.

9. Presser M, Birkhan J, Adler R, Hanani A, Eisenberg E (2000) Transcutaneous electrical nerve stimulation (TENS) during epidural steroids injection: A randomized controlled trial. Pain Clinic 12(2): 77-80.

10. Benedetti F, Amanzio M, Casadio C, Cavallo A, Cianci R, et al. (1997) Control of postoperative pain by transcutaneous electrical nerve stimulation after thoracic operations. Ann Thorac Surg 63(3): 773-6.

11. Coyne PJ, MacMurren M, Izzo T, Kramer T (1995) Transcutaneous electrical nerve stimulator for procedural pain associated with intravenous needlesticks. J Intraven Nurs 18(5): 263-7.

12. Forster EL, Kramer JF, Lucy SD, Scudds RA, Novick RJ (1994) Effect of TENS on pain, medications, and pulmonary function following coronary artery bypass graft surgery. Chest 106(5): 1343-8.

13. Lander J, Fowler-Kerry S (1993) TENS for children's procedural pain. Pain 52(2): 209-16.

14. Hargreaves A, Lander J (1989) Use of transcutaneous electrical nerve stimulation for postoperative pain. Nurs Res 38(3): 159-61.

15. Roth PM, Thrash WJ (1986) Effect of transcutaneous electrical nerve stimulation for controlling pain associated with orthodontic tooth movement. Am J Orthod Dentofacial Orthop 90(2): 132-8.

16. Conn IG, Marshall AH, Yadav SN, Daly JC, Jaffer M (1986) Transcutaneous electrical nerve stimulation following appendicectomy: the placebo effect. Ann R Coll Surg Engl 68(4): 191-2.

17. Galloway DJ, Boyle P, Burns HJ, Davidson PM, George WD (1084) A clinical assessment of electroanalgesia following abdominal operations. Surg Gynecol Obstet 159(5): 453-6.
